# Identifying differentially expressed genes and miRNAs in Kawasaki disease by bioinformatics analysis

**DOI:** 10.1038/s41598-022-26608-x

**Published:** 2022-12-19

**Authors:** Yanliang Cai, Weitao Hu

**Affiliations:** 1 Department of Pediatrics, The First Affiliated Hospital of Fujian Medical University, Fuzhou, 350000 Fujian People’s Republic of China; 2 Department of Rheumatology, The Second Affiliated Hospital of Fujian Medical University, Quanzhou, 362000 Fujian People’s Republic of China

**Keywords:** Biomarkers, Diseases, Rheumatology

## Abstract

Kawasaki disease (KD) is an acute systemic immune vasculitis caused by infection, and its etiology and underlying mechanisms are not completely clear. This study aimed to identify differentially expressed genes (DEGs) with diagnostic and treatment potential for KD using bioinformatics analysis. In this study, three KD datasets (GSE68004, GSE73461, GSE18606) were downloaded from the Gene Expression Omnibus (GEO) database. Identification of DEGs between normal and KD whole blood was performed using the GEO2R online tool. Gene ontology and Kyoto Encyclopedia of Genes and Genomes (KEGG) functional enrichment analysis of DEGs was undertaken with Metascape. Analysis and visualization of protein–protein interaction networks (PPI) were carried out with STRING and Cytoscape. Lastly, miRNA-genes regulatory networks were built by Cytoscape to predict the underlying microRNAs (miRNAs) associated with DEGs. Overall, 269 DEGs were identified, including 230 up-regulated and 39 down-regulated genes. The enrichment functions and pathways of DEGs involve regulation of defense response, inflammatory response, response to bacterium, and T cell differentiation. KEGG analysis indicates that the genes were significantly enriched in Neutrophil extracellular trap formation, TNF signaling pathway, Cytokine-cytokine receptor interaction, and Primary immunodeficiency. After combining the results of the protein–protein interaction (PPI) network and CytoHubba, 9 hub genes were selected, including *TLR8, ITGAX, HCK, LILRB2, IL1B, FCGR2A, S100A12, SPI1,* and *CD8A*. Based on the DEGs-miRNAs network construction, 3 miRNAs including mir-126-3p, mir-375 and mir-146a-5p were determined to be potential key miRNAs. To summarize, a total of 269 DEGs, 9 hub genes and 3 miRNAs were identified, which could be considered as KD biomarkers. However, further studies are needed to clarify the biological roles of these genes in KD.

## Introduction

Kawasaki disease (KD) is an acute pediatric self-limiting systemic inflammatory vasculitis that usually affects small and medium-sized blood vessels throughout the body and was first identified by Dr. Kawasaki of Japan in 1967^1,2^. KD is diagnosed mainly by clinical criteria. The diagnosis of a typical KD is based on fever lasting ≥ 5 days with 4 of the 5 main clinical features satisfied (including erythema and dehiscence of the lips, strawberry tongue, erythema of the oral mucosa; bilateral bulbar conjunctival injection without exudation; rash: maculopapular or diffuse erythema; erythema and edema of the hands and feet in the acute phase; and usually unilateral enlarged cervical lymph nodes, > 1.5 cm in diameter). Patients lacking the full clinical features of a typical KD can be considered diagnosed in most cases if a coronary artery abnormality is detected^1,3^. The cause of KD is uncertain and there is still no specific diagnostic test. Combining the results of several studies, the consensus is that KD is an immune-related disease triggered by infection in genetically susceptible patients^4–6^. Approximately 25% of untreated KD patients will result in a coronary aneurysm or even a life-threatening condition. Although prompt treatment with intravenous immunoglobulin (IVIG) reduces this risk to 3% to 5%, the side effects associated with IVIG should not be underestimated^1^. However, biomarkers can assist clinicians in characterizing the severity and prognosis of the disease in early diagnosis and intervention. Therefore, studying and discovering the precise molecular mechanisms of KD is essential for developing therapeutic strategies.

Microarray technology and bioinformatics analysis have been broadly applied to screen for genetic alterations at the genomic level and to identify differentially expressed genes (DEGs) and functional pathways involved in the development and progression of KD^7–9^. Yet, it is difficult to obtain reliable results with a single microarray analysis due to its high rate of false positives. Therefore, in this study, to identify DEGs between normal and KD blood samples, 3 mRNA microarray datasets were downloaded from Gene Expression Omnibus (GEO). Subsequently, Gene ontology (GO) and Kyoto Encyclopedia of Genes and Genomes (KEGG) functional enrichment analysis of DEGs was undertaken with Metascape. Analysis and visualization of protein–protein interaction networks (PPI) were carried out with STRING and Cytoscape. Lastly, miRNA-genes regulatory networks were built by Cytoscape to predict the underlying microRNAs (miRNAs) associated with DEGs. To summarize, a total of 269 DEGs, 9 hub genes and 3 miRNAs were identified, which could be considered as KD biomarkers.

## Materials and methods

### Microarray data collection

GEO (http://www.ncbi.nlm.nih.gov/geo)^10^ is a public functional genomics data repository of high throughput gene expression data, chips and microarrays. The GSE68004^11^ and GSE73461^12^ datasets generated using the GPL10558 platform(Illumina HumanHT-12 V4.0 expression beadchip), and GSE18606^13^ generated on the GPL6480 platform (Agilent-014850 Whole Human Genome Microarray 4 × 44 K G4112F) were downloaded from GEO. According to the annotation information in the platform, the probes are transformed into the corresponding gene symbols. Samples in this study were selected untreated or without other control factors that might influence the variables. The GSE68004 dataset contained 89 complete/incomplete KD whole blood samples and 37 control samples; the GSE73461 dataset contained 77 whole blood samples from KD patients without definite bacterial or viral infections and 55 healthy controls; the GSE18606 dataset contained 8 whole blood samples from KD patients without IVIG treatment and 9 controls.

### DEGs identification

Determining DEGs between KD and normal blood samples was performed using GEO2R (http://www.ncbi.nlm.nih.gov/geo/geo2r). GEO2R is an interactive web-based tool that allows users to compare two or more datasets in the GEO series to determine DEGs for various experimental situations. The adjusted *P*-values (adj. *P*) and Benjamini and Hochberg false discovery rate were used to strike a balance between finding statistically significant genes and limiting false positives. Genes with no corresponding gene symbols in the probe set or with more than one probe set were removed or averaged out separately. |log FC (fold change)|> 1 and adj. *P*-value < 0.01 were considered statistically significant.

### Enrichment analysis of KEGG and GO for DEGs

Metascape (https://metascape.org/gp/index.html#/main/step1)^14^ is an analytical website which incorporates functional enrichment, interactome analysis, gene annotation and membership search in a comprehensive portal leveraging more than 40 independent knowledge bases. The KEGG is a resource of databases for the clarification of high-level features and effects of biological systems^15,16^. Gene Ontology (GO) is a premier bioinformatics program for high-quality functional gene annotation based on biological processes (BP), molecular functions (MF), and cellular components (CC)^17^. Metascape was used to determine the features of DEGs with the screening criteria set to minimum overlap = 3 and minimum richness = 1.5. *P* < 0.01 was considered statistically significant.

### Construction of PPI network and analysis of module

The PPI network was constructed using the Search Tool for the Retrieval of Interacting Genes (STRING; http://string-db.org) (version 11.5)^18^ online database, and the parameters were set as follows: meaning of network edges: confidence level; minimum required interaction score: medium confidence (0.400). An open-source bioinformatics software platform, Cytoscape (version 3.9.1) is used to visualize molecular interaction networks^19^. Molecular Complex Detection (MCODE) (version 2.0) is a plug-in for Cytoscape to perform clustering of a given network according to the topology to identify densely connected regions^20^. Using Cytoscape to map the PPI network and using MCODE to identify the most important modules in the PPI network. The selection criteria were as follows: MCODE scores > 5, node score cut-off = 0.2, degree cut-off = 2, k-score = 2 and Max depth = 100.

### Hub genes selection and analysis

The overlap of the top 20 genes based on the algorithms MCC, Maximum Neighborhood Component (MNC), DNMC, Closeness, Degree, Stress, Betweenness, Bottleneck, and Edge Penetration Component (EPC) were identified as hub genes using the cytoHubba plugin. Metascape was employed to forecast the function of hub genes and the screening conditions were set to Min Enrichment = 1.5 and Min overlap = 3. *P* < 0.01 was considered to be statistically significant.

### Evaluation and validation of hub genes

ROC curves and box plots of gene expression were performed using Graphpad Prism software. When the area under the curve (AUC) exceeds 0.7, the gene will be regarded as highly diagnostic for KD. A two-sample t-test was used to compare the gene expression levels of KD and normal samples.

### MiRNAs related to hub genes

Mapping of the top 10 hub genes to their corresponding miRNAs was performed using NetworkAnalyst 3.0 (https://www.networkanalyst.ca/)^21^, an online platform for visualization that facilitates the search for miRNA-gene interactions in gene regulatory networks. Each hub gene was identified as miRNAs with a degree cutoff value = 1.0. Finally, these hub genes and miRNAs were mapped by Cytoscape 3.9.1.

## Results

### Identification of DEGs in KD

**Figure 1:**
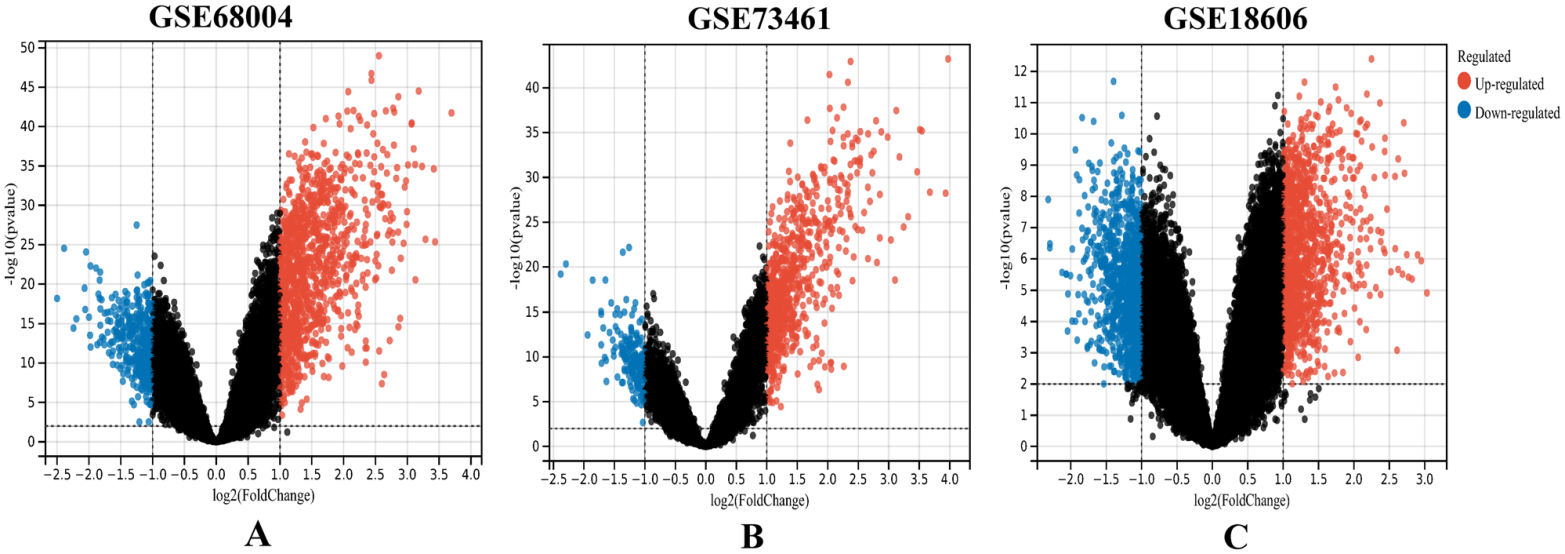
Volcano plots showing differentially expressed genes (DEGs) between the control and KD groups. (**A**–**C**) DEGs of the GSE68004, GSE73461 and GSE18606 datasets are shown, respectively. Red data points indicate up-regulated genes and blue ones indicate down-regulated genes. Genes without any significant differences are in black.

We retrieved three datasets (GSE68004, GSE73461, and GSE18606) from the GEO database which contained the gene expression profiles of KD whole blood samples and healthy controls. See Table 1 for details of these three datasets. The DEGs (1103 in GSE68004, 684 in GSE73461 and 1602 in GSE18606) were identified after normalization of the microarray outcomes. The DEGs in the GSE68004, GSE73461 and GSE18606 datasets included 832 up-regulated and 271 down-regulated genes, 554 up-regulated and 130 down-regulated genes, and 821 up-regulated and 781 down-regulated genes, respectively. DEGs were all identified by comparison of the gene expression profiles of healthy controls and KD whole blood samples. Figure 1 showed the gene expression profiles of the DEGs in the three datasets containing data from the 2 sets of samples.

**Table 1:** Details for GEO Kawasaki disease data.

Reference	GEO	Platform	Control	KD
Jaggi (2018)	GSE68004	GPL10558	37	89
Wright (2018)	GSE73461		55	77
Fury (2010)	GSE18606	GPL6480	9	8

**Figure 2:**
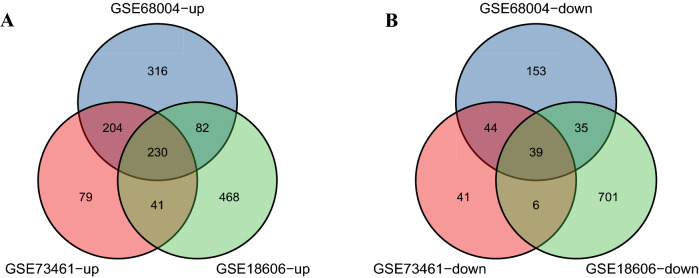
Venn diagram displaying the overlapping differentially expressed genes (DEGs) in three datasets searched from Gene Expression Omnibus (GEO). (**A**, **B**) illustrate the overlap of up-regulated and down-regulated genes in the GSE68004, GSE73461 and GSE18606 datasets, respectively.

Further screening of these genes was performed and Venn diagrams were drawn representing these genes. As shown in Fig. 2, 269 DEGs were found to be significantly differentially expressed in the 3 groups, of which 230 genes were upregulated and 39 genes were downregulated (Table 2).

**Table 2:** Screening DEGs in Kawasaki disease patients by integrated microarray.

DEGs	Gene terms
Upregulated	ACER3 ACSL1 ADCY3 ADGRE1 ADGRG3 ADM AGTRAP AIM2 ALOX5AP ALPL ANKRD22 ANPEP ANXA3 APMAP APOBR AQP9 ATP11B ATP6V1C1 ATP9A B4GALT5 BATF BCL6 BPI C19orf38 C3AR1 C5AR1 CA4 CAMP CCR1 CD55 CD58 CDA CDC42EP3 CDK5RAP2 CEACAM1 CEACAM3 CEBPB CETP CKAP4 CKLF CLEC4A CLEC4D CMTM2 CNIH4 CR1 CREB5 CRISPLD2 CSF2RA CSF3R CST7 CSTA CTSA CXCL16 CYP1B1 CYSTM1 DACH1 DGAT2 DHRS13 DRAM1 DSE DYSF ECHDC3 EIF4E3 EMILIN2 ETS2 EXOC6 EXOSC4 FAR2 FCAR FCGR1B *FCGR2A* FES FFAR2 FGR FKBP5 FLOT1 FLOT2 FLVCR2 FOLR3 FPR1 FRAT1 GADD45A GAS6 GAS7 GBA GK GNG10 GNS GRB10 GRINA GYG1 HAUS4 *HCK* HIST1H2BD HIST1H3F HIST2H2AA4 HIST2H2AC HIST2H2BE HK3 HMGB2 HPGD HSPA1A IER3 IFITM3 IGSF6 IL10RB IL18R1 IL18RAP *IL1B* IL1RN IMPA2 IMPDH1 IRAK3 *ITGAX* JUNB KCNJ2 KIF1B LCN2 LILRA2 LILRA5 *LILRB2* LILRB3 LIMK2 LMNB1 LPCAT2 LRG1 LRPAP1 LTB4R LTBR LY96 MAPK14 MCEMP1 MGAM MKNK1 MLKL MMP25 MMP9 MSRB1 MYD88 MYL9 NABP1 NACC2 NCF4 NECTIN2 NFKBIZ NLRC4 NQO2 OSCAR OSM P2RX1 PADI4 PDLIM7 PFKFB3 PFKFB4 PGD PGLYRP1 PGS1 PHC2 PHTF1 PIK3AP1 PILRA PLBD1 PLIN3 PLP2 POR PRKCD PROK2 PSTPIP2 PYGL QSOX1 RAB24 RAB27A RAB31 RAB32 RALB RETN RGL4 RNASE2 RNF144B RNF24 ROPN1L RRAGD S100A11 *S100A12* SBNO2 SEMA4A SERPINB1 SHKBP1 SIGLEC10 SIGLEC5 SIGLEC7 SIGLEC9 SIPA1L2 SIRPA SIRPD SLC11A1 SLC12A9 SLC22A4 SLC26A8 SLC2A3 SLC37A3 SMARCD3 SNX20 SOCS3 SORT1 *SPI1* ST3GAL4 STOM STXBP2 TBC1D2 TCN1 TIFA TLR5 *TLR8* TMEM120A TMEM165 TNFAIP6 TNFSF10 TNFSF13B TREML1 TSHZ3 TSPO TXN UPP1 USB1 VNN2 WSB1 WSB2 ZNF438 ZNF467
Downregulated	ABLIM1 ADGRG1 BCL11B CD2 CD3G *CD8A* CD96 EPHX2 GNLY GPR183 GZMH GZMK IL2RB IL7R KLRB1 KLRG1 LBH LCK LEF1 LRRN3 MAL NELL2 PLEKHA1 PTPN4 PVRIG PYHIN1 RORA SAMD3 SGK223 SKAP1 SPOCK2 STAT4 SYTL2 TARP TGFBR3 TMEM204 ZAP70 ZNF683 ZNF831

### Enrichment analysis of KEGG and GO for DEGs

**Figure 3:**
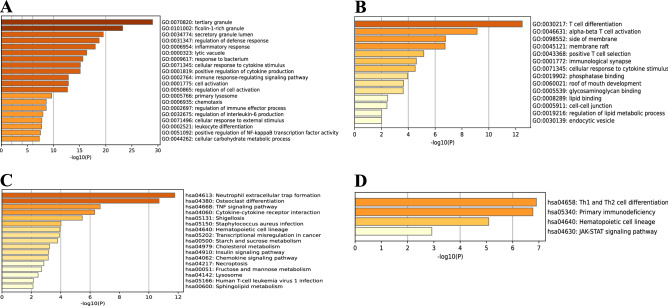
Functional enrichment of DEGs. Bar graphs display the results of the top 20 enrichment analyses for up-regulated genes (**A**, **C**) and down-regulated genes (**B**, **D**). *P*-value is shown in color.

We performed functional enrichment analysis of up-regulated and down-regulated genes in order to predict the biological functions of DEGs. GO analysis revealed that the upregulated genes were enriched mainly in tertiary granule, secretory granule lumen, regulation of defense response, and inflammatory response (Fig. 3A), while the down-regulated genes were enriched significantly in T cell differentiation, alpha–beta T cell activation, and positive T cell selection (Fig. 3B). KEGG pathway analysis revealed that the up-regulated genes were significantly enriched in Neutrophil extracellular trap formation, Osteoclast differentiation, and Cytokine-cytokine receptor interaction (Fig. 3C), while the down-regulated genes were mainly enriched in Th1 and Th2 cell differentiation and Primary immunodeficiency (Fig. 3D).

### Construction of PPI network, analysis of module, and identification of hub genes

**Figure 4:**
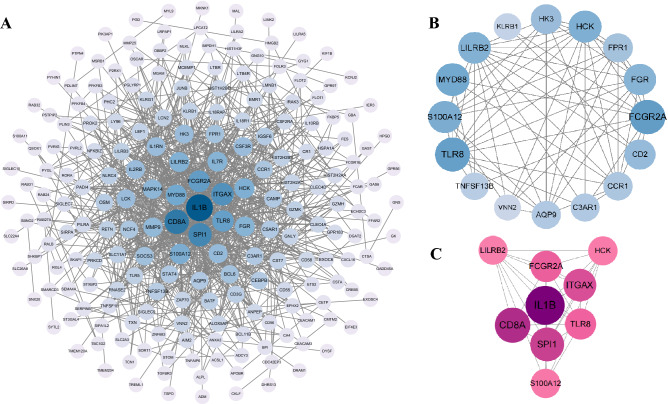
Network construction and module analysis. (**A**) Construction of a protein–protein interaction network using Cytoscape. The network includes 269 nodes and 869 edges. 2 edges between nodes indicate gene–gene interactions. The node corresponding to each gene is sized and colored according to the degree of interaction. The color scale indicates the change in the degree of each gene from high (blue) to low (white). Closer to the blue node, the higher the degree of connectivity between the 2 nodes. (**B**) The most densely connected region of the PPI network (16 nodes, 64 edges) was identified using MCODE. (**C**) 9 hub genes were determined in the densest connected region using the 8 algorithms in cytoHubba. The degree scores are represented in pink color. A darker color means a higher degree score.

The analysis of PPI for DEGs was based on the STRING database and the results were visualized with Cytoscape (Fig. 4A). With MCODE, a plug-in for Cytoscape, we determined the most densely connected regions (16 nodes, 64 edges) in the PPI network (Fig. 4B). The top 20 genes were calculated by 8 algorithms of the Cytoscape plugin cytoHubba (Table 3). Subsequently, the top 9 intersecting genes analyzed according to these 8 algorithms were selected as hub genes, which included *CD8A, FCGR2A, HCK, IL1B, ITGAX, LILRB2, S100A12*, *SPI1,* and *TLR8* (Fig. 4C).

**Table 3:** The top 20 hub genes rank in cytoHubba.

MCC	Degree	Stress	Closeness	MNC	EPC	Betweenness	Bottleneck	overlap
AQP9	CD2	CD8A	CD8A	BCL6	CCR1	CD8A	CD8A	CD8A FCGR2A HCK IL1B ITGAX LILRB2 S100A12 SPI1 TLR8
C3AR1	CD8A	FCGR2A	CSF3R	CD2	CD2	FCGR2A	CEBPB	
CCR1	CSF3R	FGR	FCGR2A	CD8A	CD8A	FGR	CSF3R	
CD8A	FCGR2A	HCK	FGR	CSF3R	CSF3R	FLOT1	FCGR2A	
CSF3R	FGR	HIST2H2BE	HCK	FCGR2A	FCGR2A	HCK	HCK	
FCGR2A	HCK	HK3	IL1B	FGR	FGR	HIST2H2BE	HK3	
FGR	HK3	IL1B	IL1RN	FPR1	FPR1	HK3	IL1B	
FPR1	IL1B	ITGAX	IL2RB	HCK	HCK	IL1B	IL1RN	
HCK	IL1RN	LILRB2	IL7R	IL1B	IL1B	ITGAX	ITGAX	
HK3	IL2RB	MAPK14	ITGAX	IL1RN	IL1RN	LILRB2	LCK	
IGSF6	IL7R	MMP9	LCK	IL2RB	IL2RB	MAPK14	LILRB2	
IL1B	ITGAX	MYD88	LILRB2	IL7R	IL7R	MYD88	MAPK14	
IL7R	LCK	NCF4	MAPK14	ITGAX	ITGAX	NCF4	NCF4	
ITGAX	LILRB2	OSM	MMP9	LCK	LCK	OSM	OSM	
LCK	MAPK14	S100A12	MYD88	LILRB2	LILRB2	S100A12	PADI4	
LILRB2	MMP9	SMARCD3	NCF4	MMP9	MMP9	SMARCD3	RETN	
MYD88	MYD88	SOCS3	S100A12	MYD88	MYD88	SOCS3	S100A12	
S100A12	S100A12	SORT1	SOCS3	S100A12	S100A12	SORT1	SMARCD3	
SPI1	SPI1	SPI1	SPI1	SPI1	SPI1	SPI1	SPI1	
TLR8	TLR8	TLR8	TLR8	TLR8	TLR8	TLR8	TLR8	

### Hub genes selection and analysis

**Figure 5:**
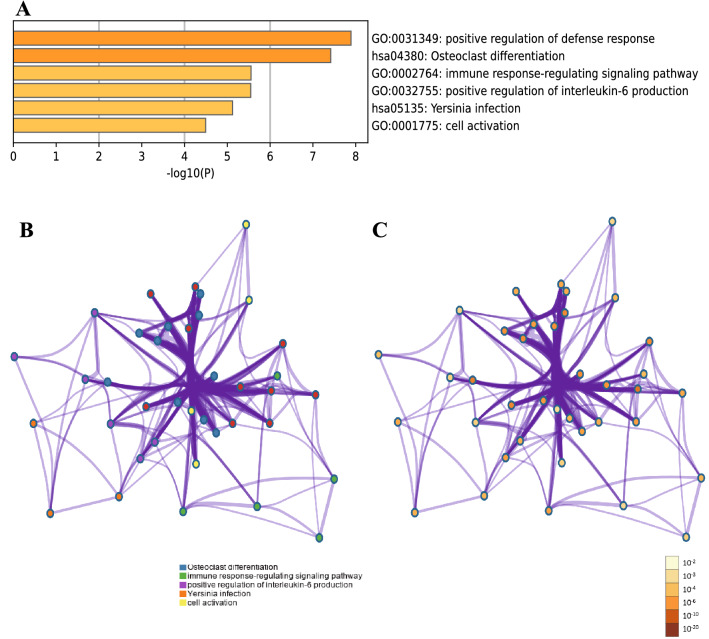
Functional enrichment of hub genes (**A**) Bar graph of GO analyses of hub genes. P-values were indicated by color. The network of enriched terms of hub genes; colors indicated the same cluster-ID (**B**) and *P*-value (**C**).

The details of symbols, abbreviations, and functions about hub genes are shown in Table 4. Functional enrichment analysis showed that the 9 hub genes were mainly concentrated in biological processes (BP), namely positive regulation of defense response, immune response-regulating signaling pathway, positive regulation of interleukin-6 production and cell activation, as well as two KEGG that are Osteoclast differentiation and Yersinia infection (Fig. 5A–C, Table 5).

**Table 4.** 9 hub genes and their functions.

Gene Symbol	Description	Function
TLR8	Toll Like Receptor 8	Key component of innate and adaptive immunity
ITGAX	Integrin subunit alpha X	A receptor for fibrinogen. It recognizes the sequence G-P-R in fibrinogen; mediates cell–cell interaction during inflammatory responses
HCK	Tyrosine-protein kinase HCK	Transmiting signals from cell surface receptors and plays an important role in the regulation of innate immune responses
LILRB2	Leukocyte immunoglobulin-like receptor subfamily B member 2	Involved in the down-regulation of the immune response and the development of tolerance
IL1B	Interleukin 1 Beta	Potent proinflammatory cytokine
FCGR2A	Fc fragment of IgG receptor IIa	By binding to IgG it initiates cellular responses against pathogens and soluble antigens. Promotes phagocytosis of opsonized antigens
SPI1	Spi-1 proto-oncogene/Transcription factor PU.1	a transcriptional activator that may be specifically involved in the differentiation or activation of macrophages or B- cells
CD8A	T-cell surface glycoprotein CD8 alpha chain	Integral membrane glycoprotein that plays an essential role in the immune response and serves multiple functions in responses against both external and internal offenses
S100A12	Protein S100-A12	a calcium-, zinc- and copper-binding protein which plays a prominent role in the regulation of inflammatory processes and immune response

**Table 5:** Functional enrichment analysis of hub genes.

Term	Description	Count in gene set	-LogP	Gene symbol
GO:0031349(BP)	positive regulation of defense response	5	7.8903641222	HCK, IL1B, S100A12, SPI1, TLR8
hsa04380(KEGG)	Osteoclast differentiation	4	7.4166437419	FCGR2A, IL1B, SPI1, LILRB2
GO:0002764(BP)	immune response-regulating signaling pathway	4	5.5546860646	CD8A, HCK, LILRB2, TLR8
GO:0032755(BP)	positive regulation of interleukin-6 production	3	5.5469520525	IL1B, LILRB2, TLR8
hsa05135(KEGG)	Yersinia infection	3	5.122458522	CD8A, FCGR2A, IL1B
GO:0001775(BP)	cell activation	4	4.4937053227	CD8A, IL1B, S100A12, SPI1

### Evaluation and validation of hub genes

**Figure 6:**
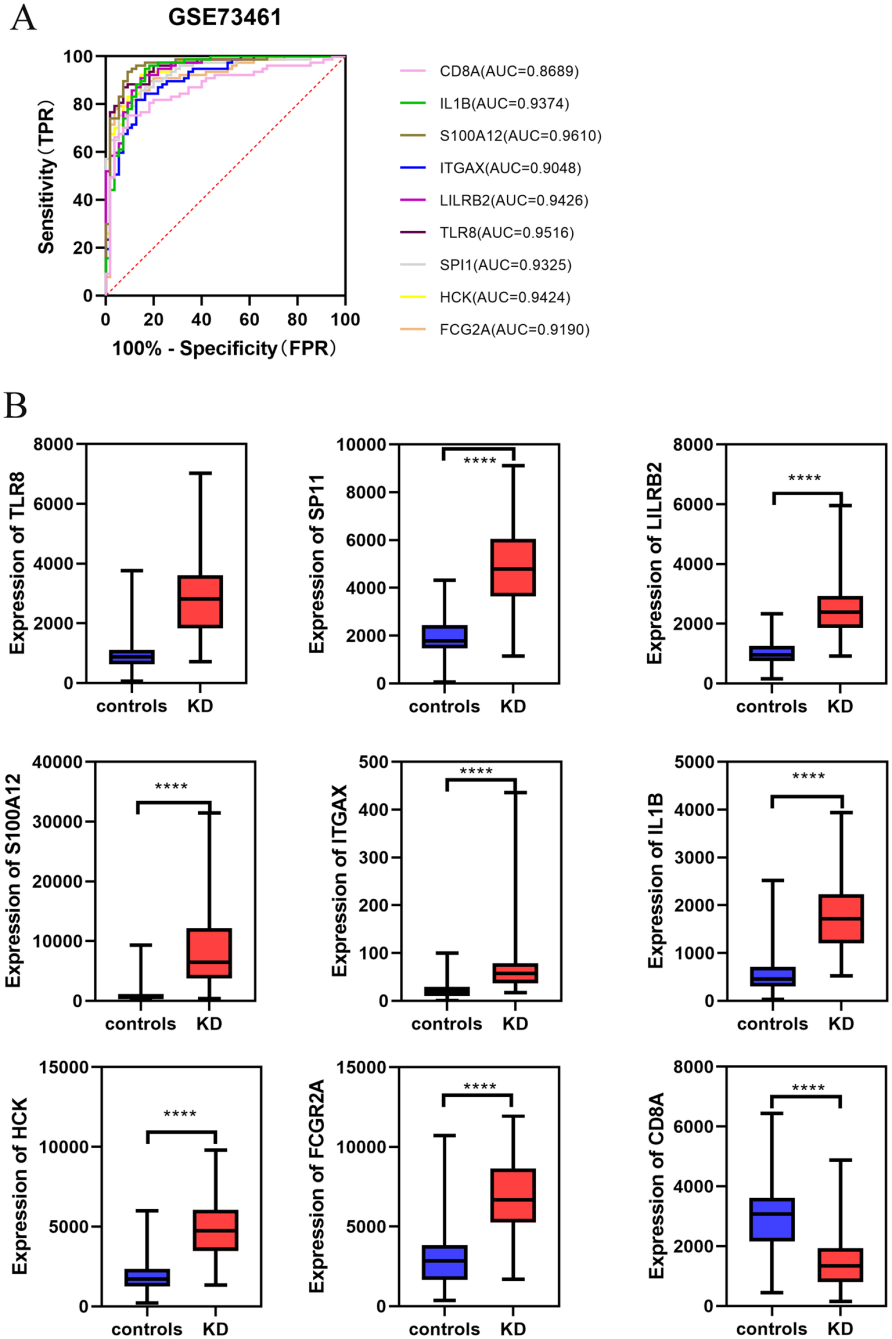
Validation of hub genes in the GSE73461 dataset. (**A**) ROC analysis of hub genes in KD. Different genes are indicated by different colors. (**B**) Box plot depicting the expression of hub genes in KD and normal samples.

**Figure 7:**
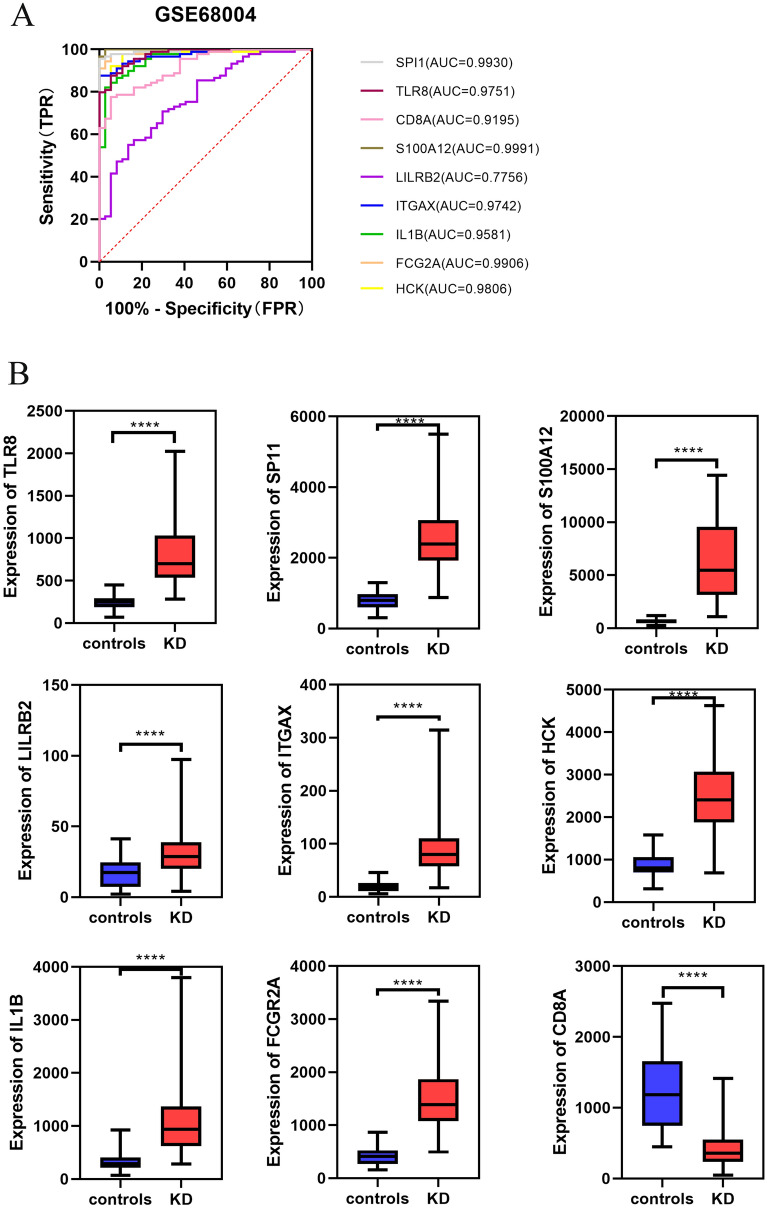
Validation of hub genes in the GSE68004 dataset. (**A**) ROC analysis of hub genes in KD. Different genes are indicated by different colors. (**B**) Box plot depicting the expression of hub genes in KD and normal samples.

**Figure 8:**
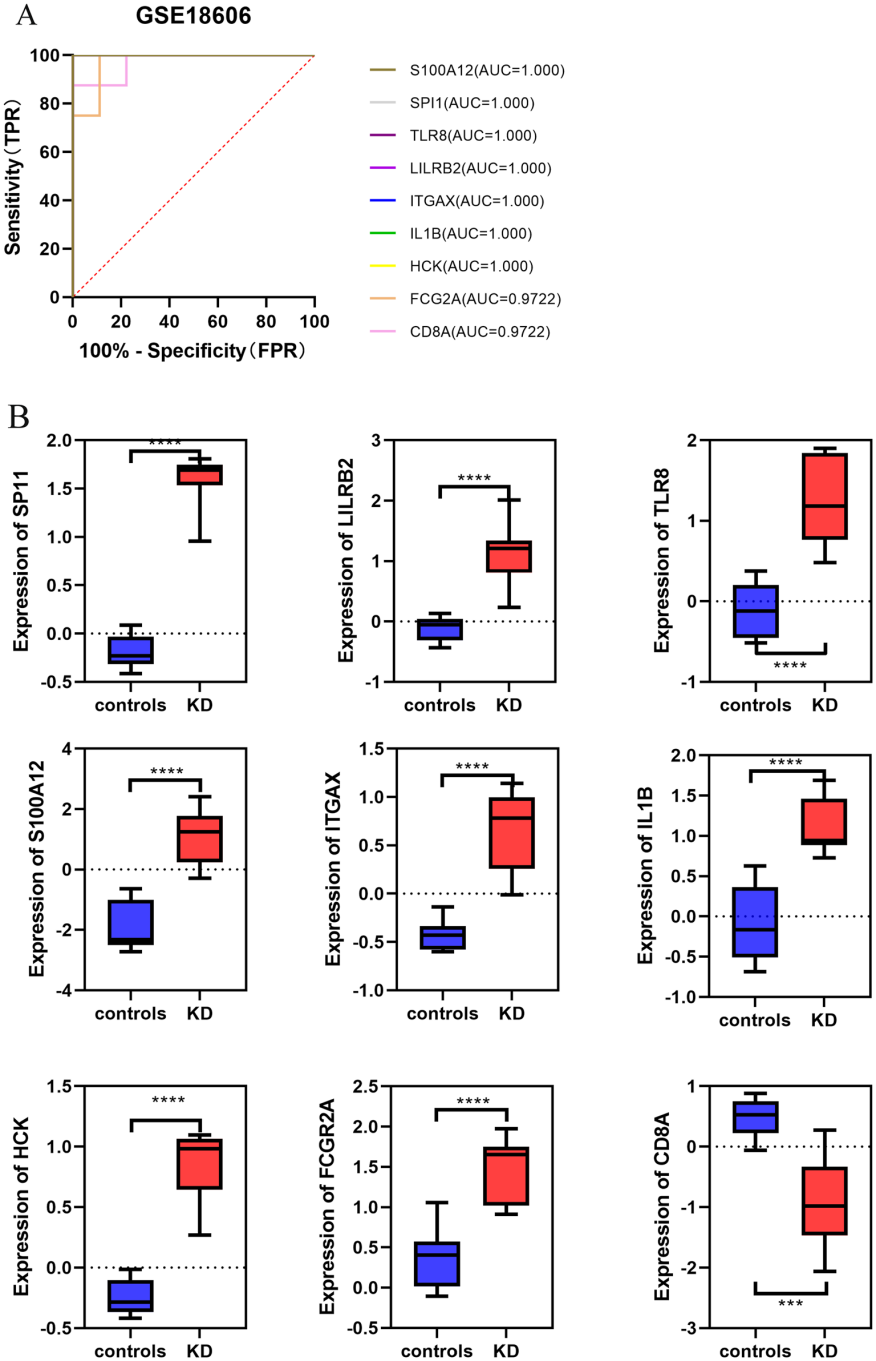
Validation of hub genes in the GSE18606 dataset. (**A**) ROC analysis of hub genes in KD. Different genes are indicated by different colors. (**B**) Box plot depicting the expression of hub genes in KD and normal samples.

The diagnostic value in KD was determined by the area under the curve of ROC analysis based on the hub genes derived from the previous step. The results showed that the AUC of all hub genes in GSE73461 was greater than 0.8 (Fig. 6A). In the dataset GSE68004, the AUC values of all genes were higher than 0.7 (Fig. 7A). In the dataset GSE18606, all genes had AUC values higher than 0.8 (Fig. 8A). It can be observed that in these datasets, the expression of all hub genes was higher and statistically significant in KD (Figs. 6B, 7B and 8B). Therefore, combining the expression levels of hub genes and ROC results, we identified the above screened hub genes as candidate markers.

### Establishment of miRNAs-hub genes regulatory network

**Figure 9:**
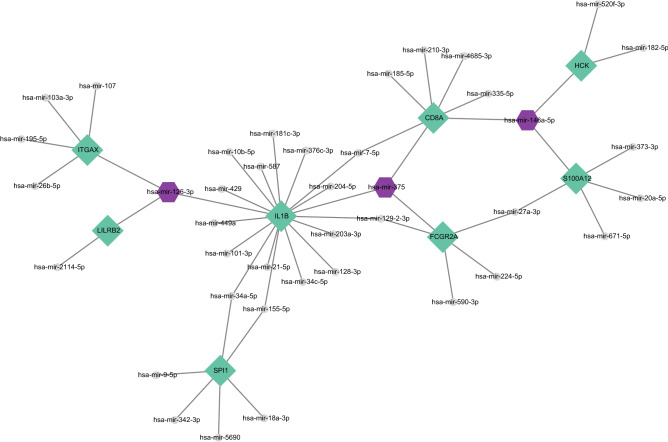
Network of integrated miRNA-DEGs with 8 hub genes. Green diamonds indicate the 8 hub genes. Gray circles indicate miRNAs with low connection to the hub genes. Dark pink octagons indicate miRNAs with high connection to the hub genes.

MiRNAs play multiple roles in the modulation of gene expression. The miRNAs and hub gene regulatory networks are built using Cytoscape to predict miRNAs targeting hub genes based on the NetworkAnalyst database. In Fig. 9, the 8 hub genes with their corresponding molecules of regulatory miRNAs are shown. One hub gene (*IL1B*) has 2 target miRNAs (mir-126-3p and miR-375). Among the 3 miRNAs, mir-126-3p was the common target of 3 hub genes (*IL1B, LILRB2* and *ITGAX*), while mir-146a-5p was the common target of 3 hub genes (*CD8A, HCK* and *S100A12*).

## Discussion

Whole-genome association studies have completely transformed the complex field of polygenic diseases and contributed to the achievement of several KD susceptibility genes, resulting in new insights into the pathogenesis of the disease. In the present study, we identified 269 DEGs, including 230 up-regulated genes and 39 down-regulated genes. The results of GO functional enrichment showed that these DEGs were enriched mainly in tertiary granule, secretory granule lumen, regulation of defense response and inflammatory response. In the PPI network of DEGs, 9 (*TLR8, ITGAX, HCK, LILRB2, IL1B, FCGR2A, S100A12, SPI1* and*CD8A*) out of 269 genes had high degree of interaction. All the 9 hub genes were up-regulated in patients with KD except for *CD8A*. The results of GO functional enrichment indicated that these 9 genes were enriched mainly in positive regulation of defense response, immune response-regulating signaling pathway, positive regulation of interleukin-6 production and cell activation, as well as two KEGG that are Osteoclast differentiation and Yersinia infection. Earlier studies have shown that the infectious trigger of KD leads to a massive activation of the immune system, causing the coronary arteries to be in a prolonged self-immune response^22^. In addition, KD presents with a marked systemic inflammatory response^23^. It has been shown that IL-6 production is significantly elevated in KD patients, suggesting that there may be an underlying immune susceptibility in KD patients^24^. Jing et al. suggested that the formation of Neutrophil extracellular trap may alter the biological response of peripheral blood mononuclear cells (PBMC) and affect vascular injury in the KD^25^. These GO terms and the enrichment results of the KEGG pathway suggest that the DEGs or hub genes detected in the present study may be involvement in KD progression through the approaches described above.

*TLR8*, a member of the toll-like receptor family, is expressed mainly in myeloid dendritic cells, neutrophils, and monocytes^26^. It has been shown that the innate immune receptor *TLR8* can be targeted by small molecule agents^27^. It has been shown that *TLR8*, a marker of macrophage M2, is significantly elevated in the acute phase of KD, suggesting that activated macrophages are a key driver of vasculitis in KD^28^. In our study, *TLR8* acted as an up-regulated gene and therefore we speculated that it could serve as a potential innate immunotherapeutic target for KD. *IL1B* (Interleukin 1 Beta) is a crucial mediator in the inflammatory response and is important for both hosting responses and defending against pathogens^29^. It has been shown that IL-1β plays a key role in KD -associated abdominal aortic aneurysms, and the use of IL-1R blockers that inhibit this pathway may be a promising therapeutic target^30^. In addition, Porritt et al. demonstrated that IL-1β may play a core role in mediating gender-based differences in KD, with important implications for the use of anti-IL-1β therapies for the treatment of male and female KD patients^31^. *S100A12*, a granulocyte-derived receptor for advanced glycosylation end products (RAGE) and Toll-like receptor 4 (TLR-4) agonist, has been demonstrated to be upregulated in KD as well as involved in aseptic inflammatory activation of coronary endothelial cells in KD^32,33^.

*ITGAX* is a receptor for fibrinogen that mediates cellular interactions during inflammation^34^. To date, the role of *ITGAX* in KD is still unknown, and although one study has demonstrated that it is the key gene in KD^35^, further studies are needed to determine it. Hematopoietic cell kinase (*HCK*) is a member of the SRC family of cytoplasmic tyrosine kinases (SFKs) that are expressed in cells of the myeloid and B-lymphocyte lineages and may serve as therapeutic targets in immune cells and cancer cells^36^. Leukocyte immunoglobulin-like receptor subfamily B member 2 (*LILRB2*, also known as Ig-like transcript 4) is a receptor for class I MHC antigens that identify a broad range of HLAs and could participate in the immune response^37^. HLA class II has been shown to influence the genetic risk of KD through genetic polymorphisms^38^, suggesting that *LILRB2* may be involved in the genetic susceptibility to KD. *FCGR2A* (Fc fragment of IgG receptor IIa, also known as CD32) encodes cell surface receptor protein discovered on phagocytes and participates in phagocytic clearance of antigen–antibody complexes^39^. Earlier studies have identified *FCGR2A* as a genetic locus associated with KD susceptibility^40^. Dysregulated B-cell signaling (such as *FCGR2A*) in genetic risk factors has been shown to increase susceptibility to KD^41^.

The T-cell surface glycoprotein CD8 alpha chain (*CD8A*), as an integral membrane glycoprotein, plays an important role in the immune response and has multiple functions in the response against external and internal attacks^42^. The role of CD8A in KD remains unreported, and interestingly, it was the only down-regulated hub gene in our study, suggesting a possible protective role in the progression of KD. Transcription factor PU.1 (*SPI1*), is a transcriptional activator that may be specifically involved in the differentiation or activation of macrophages or B cells^43^. Several bioinformatics studies have identified a correlation between *SPI1* and KD^35,44^, but the exact mechanism remains to be explored.

In our study, the roles of *TLR8, S100A12* and *IL1B* in KD have been validated through numerous previous studies, and they may serve as potential immune targets for the treatment of KD. The roles of *ITGAX, HCK, LILRB2* and *FCGR2A* in KD have not been widely studied, but based on our findings, except for *CD8A*, they were all hub genes that significantly upregulated in KD and might be used as diagnostic markers to predict disease progression.

MiRNAs are a type of small non-coding RNA that modulates mRNA expression, and they are becoming key genes in a range of cellular processes, including apoptosis, inflammation, and innate immune responses^45^. Moreover, several studies have shown that miRNAs can be involved in the disease progression of KD^46–49^. Ning et al.^50^ suggested that miR-126-3p might be a good reference miRNA gene in platelets of KD patients. It has been demonstrated that IL-10 reduces cardiovascular inflammation by interacting with pathways such as miR-375^51^, therefore it is speculated that mir-375 may be involved in KD by protecting against cardiovascular inflammation. A study showed that the genetic polymorphic locus of mir-146a was associated with susceptibility to KD in Chinese children^52^. Nevertheless, the role of these miRNAs in KD that we have identified still needs to be further explored. In addition, there are still limited studies related to genes and miRNAs in the KD.

**Figure 10:**
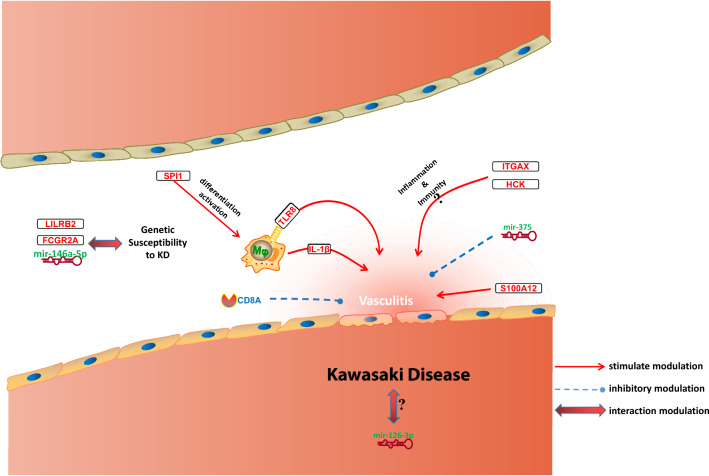
Hub genes and miRNAs in KD.

It is evident that gene-miRNA regulatory networks play a key part in the development of KD. In this way, it increases the knowledge of KD identification and contributes to targeted therapeutic management strategies and KD prediction. This study has several limitations. For one, the results of microarray expression profiling were performed with bioinformatics analysis and were not confirmed with basic experiments. Furthermore, the detailed mechanisms of how hub genes and miRNAs regulate KD deficiency were not explored. We have mapped a proposed mechanism for the main results of this study (Fig. 10). Unfortunately, there are currently very few experimental studies and related drug development for these potential biomarkers, making it difficult to explore them in more depth in conjunction with clinical data and experiments, which leaves our hypotheses without strong support. For future work, experimental validation of these findings will be performed in vitro and in vivo. There is also a need to propose effective strategies for in-depth clinical validation, e.g., increasing follow-up time to validate results, using methods including multiple regression models to confirm and increase the specificity and sensitivity of biomarkers, etc.

## Conclusion

To summarize, a total of 269 DEGs, 9 hub genes (*TLR8, ITGAX, HCK, LILRB2, IL1B, FCGR2A, CD8A, SPI1* and *S100A12*) and 3 miRNAs (mir-126-3p, mir-375 and mir-146a-5p) were identified, which could be considered as KD biomarkers. However, further studies are needed to clarify the biological roles of these genes in KD.

## Data Availability

The datasets generated and/or analyzed during the current study are available in the [GEO] repository, [https://www.ncbi.nlm.nih.gov/geo/query/acc.cgi?acc=GSE68004/GSE73461/GSE18606].
